# A Recombinant Turkey Herpesvirus Expressing F and HN Genes of Avian Avulavirus-1 (AAvV-1) Genotype VI Confers Cross-Protection against Challenge with Virulent AAvV-1 Genotypes IV and VII in Chickens

**DOI:** 10.3390/v11090784

**Published:** 2019-08-25

**Authors:** Krzysztof Śmietanka, Jolanta Tyborowska, Monika Olszewska-Tomczyk, Katarzyna Domańska-Blicharz, Zenon Minta, Lukasz Rabalski, Anna Czarnota, Krzysztof Kucharczyk, Boguslaw Szewczyk

**Affiliations:** 1 Department of Poultry Diseases, National Veterinary Research Institute, Al. Partyzantów 57, 24-100 Puławy, Poland; 2 Laboratory of Recombinant Vaccines, Intercollegiate Faculty of Biotechnology of University of Gdansk and Medical University of Gdansk, Abrahama Str. 58, 80-307 Gdansk, Poland; 3 BioVectis Ltd., Pawińskiego 5A, 02-106 Warsaw, Poland

**Keywords:** Newcastle disease, avian avulavirus 1, recombinant vector vaccine

## Abstract

Newcastle disease (ND) is responsible for significant economic losses in the poultry industry. The disease is caused by virulent strains of Avian avulavirus 1 (AAvV-1), a species within the family Paramyxoviridae. We developed a recombinant construct based on the herpesvirus of turkeys (HVT) as a vector expressing two genes: F and HN (HVT-NDV-F-HN) derived from the AAvV-1 genotype VI (“pigeon variant” of AAvV-1). This recombinant viral vaccine candidate was used to subcutaneously immunize one group of specific pathogen-free (SPF) chickens and two groups of broiler chickens (20 one-day-old birds/group). Humoral immune response was evaluated by hemagglutination-inhibition test and enzyme-linked immunosorbent assay (ELISA). The efficacy of the immunization was assessed in two separate challenge studies performed at 6 weeks of age with the use of virulent AAvV-1 strains representing heterologous genotypes IV and VII. The developed vaccine candidate elicited complete protection in SPF chickens since none of the birds became sick or died during the 2-week observation period. In the broiler groups, 90% and 100% clinical protection were achieved after challenges with AAvV-1 of IV and VII genotypes, respectively. We found no obvious relationship between antibody levels and protection assessed in broilers in the challenge study. The developed recombinant HVT-NDV-F-HN construct containing genes from a genotype VI AAvV-1 offers promising results as a potential vaccine candidate against ND in chickens.

## 1. Introduction

Newcastle disease (ND) is a highly infectious and contagious disease of birds caused by virulent strains of Avian orthoavulavirus 1 (AAvV-1), members of the family Paramyxoviridae [1]. The virus contains non-segmented, single-stranded negative-sense RNA genome encoding for 6 structural proteins: fusion (F), nucleoprotein (NP), matrix (M), phosphoprotein (P), RNA polymerase (L), haemagglutinin-neuraminidase (HN) and additionally two proteins (V and W) that are produced as a result of RNA editing of the P transcript [2]. The role of two surface glycoproteins F and HN is to mediate virus attachment, entry to the cell, and release from the cell [3]. They are also principal antigens inducing protective immune response in birds [3]. Based on genome size, AAvV-1 have been divided into two classes: class I and class II [4]. Class I viruses are predominantly of low virulence, mostly restricted to wild birds and show little genetic variation within the class. On the other hand, class II viruses are widespread in poultry, exhibit a broad range of virulence and consist of at least 18 genotypes and multiple sub-genotypes [5]. All AAvV-1 belong to a single serotype but based on the reactivity with a panel of monoclonal antibodies, antigenic variation has been observed [6]. Newcastle disease is listed in the Terrestrial Animal Health Code of the World Organisation for Animal Health but only viruses that fulfill specific virulence criteria are notifiable [7].

The disease has a worldwide distribution with some endemic areas located mostly in Africa and Asia [8]. Occasional epidemic waves in Europe, North America or Australia resulted in serious economic losses. For example, the outbreak of velogenic ND in the United States in 2002–2003 led to the culling of >3 million birds and the cost of eradication exceeded $120 million [9].

The successful prevention of ND relies on the combination of good biosecurity practices and vaccination of poultry. The major shortcomings related to the use of conventional vaccines include: failure to prevent infection and shedding, interference with maternally-derived antibodies (MDA), necessity for multiple vaccine administrations to achieve a satisfactory level of immunity, risk of adverse reactions (live vaccines), and difficulty differentiating infected from vaccinated birds [10]. To overcome these disadvantages, new-generation vaccines have been developed, including vector vaccines based on the herpesvirus of turkeys (HVT) as a backbone to express gene(s) of AAvV-1 encoding for immunogenic proteins. A commercialized vector vaccine rHVT-ND expressing the F gene of AAvV-1 has been shown to elicit clinical protection against virulent Newcastle disease in specific pathogen-free (SPF) chickens, commercial broilers, laying hens and turkeys [11,12,13,14,15].

Despite the substantial advances in hatchery vaccinations, there is still a need for improvements in immunoprophylaxis of economically important poultry diseases, especially in the face of dynamically growing global poultry population, increasing at a rate of approximately 25% per decade and expected to be the biggest meat sector by 2020 [16,17]. Therefore, the aim of the present study was to evaluate the immune response after vaccination of 1-day-old SPF and broiler chickens with a newly developed vector HVT-ND-F-HN vaccine candidate expressing the F and HN genes of a virulent genotype VI AAvV-1 and to assess the level of clinical protection against heterologous challenge with two virulent AAvV-1 strains (genotypes IV and VII). The potential advantage of the proposed solution is to provide a broader range of protection be adding two genes (instead of one) derived from an AAvV-1 genotype VI that is genetically closer to currently circulating field strains than classical vaccine strains based on genotypes I and II [5].

## 2. Materials and Methods

### 2.1. Cells and Viruses

The viruses used in the study were: HVT FC-126 (ATCC® VR-584B™), AAvV-1/pigeon/332/05 (repository of NVRI, Pulawy) used for the production of recombinant and velogenic strains AAvV-1/chicken/Poland/Radom/70 (repository of NVRI, Pulawy) and AAvV-1/chicken/Sweden/1997 (kindly provided by Dr. Siamak Zohari, National Veterinary Institute, Uppsala, Sweden) used for challenge purposes. All strains were propagated in the allantoic cavity of embryonated SPF chicken eggs (AAvV-1) or chicken embryo fibroblast (CEF) culture (HVT). The strain AAvV-1/pigeon/332/05 belongs to genotype VI (“pigeon variant” of AAvV-1) has the intracerebral pathogenicity index (ICPI) value of 1.05 and the putative F0 cleavage site sequence ^112^R-R-Q-K-R-F^117^ (typical of virulent strains). The challenge strain AAvV-1/chicken/Poland/Radom/70 belongs to genotype IV, has deduced amino acid sequence at the F protein ^112^R-R-Q-R-R-F^117^ (typical of virulent strains) and the ICPI of 1.88. The second challenge strain, AAvV-1/chicken/Sweden/1997 belongs to genotype VIIb and possesses identical F protein cleavage site as AAvV-1/chicken/Poland/Radom/70 and a similar value of ICPI of 1.86 [18]. Prior to experimental infections, both virus strains were titrated in SPF embryonated eggs.

CEF cells were isolated from 10-day-old SPF chicken embryos and cultured in Eagle’s minimal essential medium (EMEM) + 5–10% FBS. They were grown in CO_2_ incubator at 37 °C in TC flasks for 5–6 passages. They were split 1:3 once a week for running experiments using trypsin-EDTA (Sigma, St. Louis, MO, USA) diluted 1:2 in PBS.

Recombinant virus HVT-NDV-F-HN is strongly cell associated and has to be used in cell-associated form for propagation and for vaccination. For storage of recombinant virus infected CEF cells were collected 72 hours after infection and frozen in EMEM + 10% FBS containing 10% DMSO. For vaccination purposes, the recombinant virus (3rd passage) was grown in CEF cells (2nd–3rd passage). The infected cells were collected 48 hours after infection by trypsynization and cryopreserved in EMEM + 10% FBS containing 10% DMSO at the density 5 × 10^6^ cells/mL.

### 2.2. Cloning of AAvV-1/Pigeon/332/05 HN and F Genes for the Construction of Expression Cassettes

Genes coding for AAvV-1/pigeon/332/05 HN and F proteins were obtained by reverse transcription polymerase chain reaction (RT-PCR). PCR forward primers carried RE sites for SalI and reverse for HindIII. Primers for HN:

HN-SalI-FOR 5’AAAAGTCGACATGGGCTCCAACCCCTA and

HN-HindIII-REV 5’AAAAAAGCTTCTAACCAGATCTAGCTTCTTAAACC.

Primers for F: F-SalI FOR 5’AAAAGTCGACATGGGCTCCAACCCCTA 3’and

F-HindIII REV 5’GCTCAAGCTTTCATGTTCTTGTAGTGGCTCTC 3’. Amplified F and HN sequences were cloned into pJet1.2 vector (CloneJET PCR Cloning Kit, Thermo Fisher Scientific, Waltham, MA, USA) and sequenced for confirmation of correct sequences.

To obtain the expression cassette containing HN gene under EF-1a promotor with BGH pA, the HN was cut out from pJet with BglII and inserted into BglII site of pBudCE4.1 (Invitrogen, Carlsbad, CA, USA). For obtaining expression cassette with F gene under ECMV promotor (hCMV promoter with hCMV IE enhancer) and with SV40 pA, the F gene was cut out with SalI, HindIII blunted and inserted into blunted pEGFP-C1 vector (Clontech, Mountain View, CA, USA) lacking GFP by NheI and XhoI digestion. The expression plasmids were sequenced and the expression of F and HN genes was verified after transfection of CEF cells and in situ immunodetection (IPMA-immunoperoxidase monolayer assay) using chicken anti-NDV serum.

For transfer of expression cassettes into recombination transfer vector, the expression cassettes were amplified by PCR from pBud4.1-HN-NDV using primers

FpBudEF1-PacI 5’GCGATCTTAATTAAGTGAGGCTCCGGTGCCCGTCAGTGGGC3; RpBudBGHpA-RsrII 5’CGCTTCGGTCCGCATAGAGCCCACCGCATCCCCAGCATGCCT and from pE-C1-F-NDV:

FpEC1CMV-RsrII 5’GCACTCGGACCGATAGTAATCAATTACGGGGTCATTAGT;

RpEC1SV40pA SwaI 5’GCCGCATTTAAATCGCGTTAAGATACATTGATGAGTTTGGACA. The added RE sites were necessary for joining two expression cassettes carrying F and HN genes and for insertion into transfer plasmid. The amplified cassettes were cloned into pJet1.2 for different cloning options. The vectors were sequenced and expression was tested again on transfected CEF cells. To make double cassette recombination transfer vector, HN expression cassette was cut out from pJet with PacI and RsrII and inserted into compatible PacI, RsrII sites in synthetic recombination transfer vector pHV047-49 (ArtGene, Thermo Fisher Scientific, Waltham, MA, USA), and F carrying expression cassette was cut out from pJet with RsrII while SwaI and was inserted behind HN into RsrII, SwaI sites. The whole double expression cassette (with HN under EF-1a promotor and F under ECMV promotor) was cut out from pHV047-NDV-HN-F with PacI and SwaI, blunted, and cloned into SwaI site of recombination transfer vector pHV052-54 obtaining pHV052-NDV-HN-F. The synthetic recombination transfer vector pHV052-54 (ordered from GeneCust) contains homologous HVT sequences HV052-HV053 and HV054 with SwaI site for insertion of expression cassettes inside a non-coding sequence between HV053 (UL45) and HV054 (UL46) and PacI sites added on both external sides of HVT sequences. For transfection, the recombination vector was digested with PacI, the reaction mixture was denatured, and the digestion was checked on gel.

### 2.3. Generation of the Recombinant HVT-NDV-HN-F Virus

For generation of recombinant HVT-NDV-HN-F virus, the third passage of CEF grown in a 12-well plate was infected with parental HVT F-126 (MOI 0.01). 4 days after infection when cytopathic effect was well visible but cells were still alive, the medium was changed for fresh and cells were transfected using 3 µL TransIT X2 reagent (Mirus Bio, Madison, Wi, USA) mixture with 1 µg DNA of pHV052-NDV-HN-F (digested wiht PacI) according to the manufacturer’s instruction. As a control of transfection efficiency, the vector carrying EGFP pHV052-F-GFP (based on the pHV052-54 vector containing double expression cassette (with NDV F gene under EF-1a promotor and EGFP under ECMV promotor) was used. Transfected cells were daily controlled observing EGFP expression in a confocal microscope. When EGFP positive viral-like foci of cells appeared, transfected cells were delicately trypsinized, mixed with fresh non infected cells and further propagated for 3 days in 6-well plates in a few dilutions, then cells were again trypsinized, mixed with fresh cells, and propagated in 75 cm^2^ flasks; a small volume of cells was grown for 3 days in 12-well plates for immunodetection of HVT recombinants expressing NDV proteins.

### 2.4. Immunodetection and Purification of Recombinant HVT-NDV-HN-F Virus

Detection of the recombinant HVT-NDV-HN-F virus was performed by in situ immunochemical staining (IPMA: immunoperoxidase monolayer assay) of cells fixed with ice-cold acetone-methanol (1:1) for 5 min., washed 2x with PBS and blocked with 5% FBS in PBS containing 0.3% Tween 20 for 30 min. Cells were incubated for 45 min. with mixture of 3 different chicken sera anti-NDV LaSota 1:400 in the above buffer, washed 3× with PBS + 0.5% Tween 20, incubated for 45 min with 1:1000 HRP conjugated goat anti-chicken IgG (Jackson ImmunoResearch, Philadelphia, PA, USA), washed three times with PBS + 0.5% Tween 20 and with PBS only. Plaques were visualized with HRP substrate 1% AEC (3-amino-9-ethylocarbazole, Sigma) in 0.05 M sodium acetate pH 5.0 + 0.05% H_2_O_2_. They were stained for 30 min and observed using a confocal microscope. On the basis of IPMA recombinant plaques detection and calculation of their number versus unstained wild plaques, the number of HVT-NDV-HN-F infected cells was low. To concentrate them and accelerate the purification of rHVT from wt HVT, the infected cells were sorted by FACS after immunostaining (using BD FACS Calibur flow cytometer). FACS was performed using the mixture of inactivated 3 different chicken sera anti-NDV LaSota (produced in NVRI, Puławy) and Alexa-fluor 488 conjugate goat anti chicken IgG (Invitrogen). The sorted positive cells were mixed with fresh CEF and split for two wells in a 6-well plate for virus propagation and one in a 12-well plate for IPMA control. Virus was purified after few rounds of subsequent titrations in 96-well plates. The purified virus was highly cell-associated and has to be propagated by co-culturing of infected cells with fresh CEF and had to be stored cryopreserved in cell-associated form.

### 2.5. Western Blot Analysis of the Recombinant HVT-NDV-HN-F Virus

The expression of HN and F in HVT-NDV-HN-F infected CEF was tested by Western blotting using cell lysates obtained 3 days post infection (done in parallel) with recombinant and wild-type (wt) HVT viruses MOI 0.5 in 25 cm^2^ flasks. Infected cells were washed twice with PBS and lysates of cells in duplicate were subjected to 8% sodium dodecyl sulfate polyacrylamide gel electrophoresis (SDS-PAGE) and electroblotted to PVDF membrane. Membrane was blocked in 5% skimmed milk in TBS-T for 1 hour and cut for separate detection of HN and F proteins with rabbit polyclonal sera specific for HN and F of NDV: AAvV-1 LaSota and AAvV-1/pigeon/332/05 which were prepared in house. Membranes were incubated in specific rabbit sera diluted 1:500 and alkaline phosphatase goat anti-rabbit IgG conjugate (Abcam, Cambridge, UK) diluted 1:2000. Protein bands were visualized by NBT/BCiP alkaline phosphatase substrate.

Monospecific rabbit antisera were raised against single F and HN proteins produced in baculovirus system. The proteins were purified in highly denatured forms by elution from PVDV membranes after transfer from SDS-PAGE gels [19]. The F protein containing His tag was also purified in denaturating conditions on nickel-affinity column. The specificity of sera was tested by Western blotting at consecutive stages of antigen preparation.

### 2.6. Sequencing of the Recombinant HVT-NDV-HN-F Virus

CEF cells were infected with HVT-NDV-HN-F and wt HVT in 25 cm^2^ flasks. Three days post infection cells were collected, washed with PBS and whole genomic DNA was isolated using a Eurex Genomic DNA Isolation Kit. Whole genome of HVT-NDV-HN-F was sequenced using next generation platform MiSeq sequencer (Illumina, San Diego, CA, USA). A sequencing library was prepared using Nexters XT (Illumina, USA) library preparation kit as per the manufacturer’s instructions. Sequencing reads were de novo assembled using Geneious software (Biomatters Limited, Auckland, New Zealand) into single consensus sequences. Correctness of the assembled genome was further confirmed by comparison to sequences of HVT FC-126 (ATCC® VR-584B™)—parental virus and expression cassettes.

### 2.7. Genetic Stability of the Recombinant HVT-NDV-HN-F Virus

In an attempt to determine the stability of the recombinant HVT-NDV-HN-F virus during multiple passages on CEF cells, we examined the effects of virus passages on its titer as well as the presence of inserted HN and F genes of AAvV-1/pigeon/332/05 in the HVT genome. When the CEF cells monolayer reached 80% confluence in 75 cm flasks, it was infected with HVT-NDV-HN-F. Flasks were examined daily for cytopathic effects and four days later, when the effects of infection were observed on 75% of CEF surface, the culture fluids were carefully poured and infected cells harvested by scraping. The cells were suspended in culture media, divided into aliquots and stored in liquid nitrogen. These experiments were repeated 10 times. The titers of each viral stocks were determined by a limiting-dilution method with CEF cells as described earlier. Total RNAs from them were also isolated using RNeasy Mini Kit (Qiagen, Hilden, Germany) and the presence of three genes fragments were measured in RRT-PCR with following primers and probes 332-HN-F (TGCATACCAAGAACACCTG),

332-HN-R (AACAGTAGTGGGTAGCAC) and

332-HN-pro (FAM-ACTACAGGATCAGGCTGCACTCG-TAMRA);

332-F-F (TGTACAAGGATAGTGACATTC),

332-F-R (TGCGCCTTCAGTCTTTGA) and

332-F-pro (FAM-CTGAGCGGCAATACATCAGCCTG-TAMRA) and

HVT-F-2 (ATTCGCCAACCTGAACGA), HVT-R (CCAAACGTCCGTAGACGAAT) and

HVT-pro (FAM-GAAGCTGGCTTTCCTCGAATCGG-TAMRA) for inserted HN and F genes of AAvV-1/pigeon/332/05 as well as ILS region of HVT, respectively. The RT-PCR in a reaction mixture recommended by the supplier of the QuantiTect Probe RT-PCR Kit (Qiagen). Amplification was done by an initial incubation at 95 °C for 15 min, followed by 40 cycles of incubation at 95 °C for 10 sec, 57 °C for 30 sec and 72 °C for 10 sec.

### 2.8. Birds

One-day-old SPF chickens were hatched in our laboratory from SPF embryonated eggs (VALO BioMedia). One-day-old commercial broiler chickens (Ross 308) were also hatched in our facility from embryonated eggs purchased from a commercial hatchery. Twenty randomly selected broilers from the same maternal flock were bled at 1 day of age to assess the level of MDA. After hatching and immunization, the birds were kept in open cages (SPF chickens) or on deep litter (broilers) at BSL-3 facility until the challenge with the virulent (velogenic) AAvV-1 strain. Prior to challenge, SPF birds were placed in isolators equipped with HEPA filters (Montair Process Technology B.V.) located in the same BSL-3 facility while broilers remained on litter for welfare reasons. All experiments were approved by the Local Animal Ethics Committee in Lublin (permission numbers: 51/2012 and 13/2015).

### 2.9. Experimental Design

Experimental studies were carried out in three experiments (Table 1). Experiments I and II investigated the protective efficacy of the vaccine candidate against challenge with AAvV-1 genotype IV and were carried out in SPF (Trial I) and commercial broiler chickens (Trial II). The experiment in which the birds were infected with AAvV-1 genotype VII was only performed in broilers (Trial III). The birds were divided into 6 groups (20 birds in each group): two groups of SPF chickens (experimental and control group, Trial I), and four groups of commercial broiler chickens (two experimental groups and two control groups, Trial II and III). For the purpose of immunization, the rHVT- NDV-F-HN cryopreserved in liquid nitrogen was used. Before inoculation, the material was diluted 1:10 and mixed for 15 min at room temperature. The experimental birds were immunized subcutaneously at 1 day of age with 0.2 mL of the prototype vaccine (10^6.0^TCID_50_/dose). Control birds were inoculated subcutaneously with 0.2 mL of PBS. The blood was collected from SPF chickens at 2, 3, 5 and 6 weeks post vaccination (wpv) and 2 weeks post challenge (wpc) (8 wpv) while broilers were sampled at day 0 (to measure the level of MDA) and then 3 (with the exception of birds from Trial III), 5, 6 wpv and also 2 wpc (8 wpv). At 6 wpv the birds were infected intraocularly and intranasally with a dose of 10^6^EID_50_ (in 0.1 ml) of the virulent AAvV-1 strains: genotype IV (Trial I and II) and genotype VII (Trial III). After the challenge, the birds were observed for 2 weeks. Oropharyngeal and cloacal swabs (COPAN) were collected at 3, 7, 10 and 14 days post challenge (dpc) to assess the level of virus shedding.

### 2.10. Assessment of Virus Shedding

The quantification of challenge virus shed by the vaccinated and non-vaccinated birds was performed by quantitative real-time RT-PCR (qRRT-PCR). Swab samples were immersed in viral transport medium and stored at −80 °C until use. Total RNA was extracted from 0.2 mL of the medium using Qiagen RNeasy Mini Kit (Qiagen) according to the manufacturer’s protocol. The qRRT-PCR was performed on an ABI 7500 (Applied Biosystems) using Quantitect Probe PCR kit (Qiagen) in a volume of 25 µL. Primers and probe specific to the matrix gene were originally designed by Wise et al. [20] but the probe sequence was modified by Cattoli et al. [21]. The following cycle conditions of the RT and PCR were applied: 50 °C for 30 min, 95 °C for 15 min, followed by 40 cycles at 95 °C for 10 sec, 56 °C for 30 sec, and 72 °C for 10 sec. Ten-fold serial dilutions from the AAvV-1/chicken/Poland/Radom/70 and AAvV-1/chicken/Sweden/1997 strains with known titers were tested to generate a standard curve. The results were expressed as equivalent EID_50_ (eqEID_50_) per milliliter of swab medium.

### 2.11. Serological Examination

HI test using the LaSota strain as antigen was used according to recommended protocol (OIE, 2012) for testing of sera collected from immunized and control chickens. IDScreen^®^ ND Indirect (IDVet) enzyme-linked immunosorbent assay (ELISA) according to producer’s instruction was used in parallel.

### 2.12. Statistical Analysis

The survival analysis for vaccinated and control chickens for experimental trials 1–3 was carried out using the Kaplan–Meier method. The Mantel–Cox log-rank test was used to compare survival curves between the groups. Differences in challenge virus shedding between experimental and control groups were analyzed separately at various time points: 3 dpc (SPF chickens) and 3, 7 dpc (broilers), independently for oral and cloacal swabs, using a Mann–Whitney U test in STATISTICA software, version 10 (StatSoft, Inc.). *P*-value < 0.05 was considered significant.

## 3. Results

### 3.1. Construction of Recombinant Virus HVT-NDV-F-HN

Recombinant virus HVT-NDV-F-HN was obtained by the homologous recombination of parental HVT F-126 virus genome with a transfer vector carrying HVT homologous sequences HV052-HV053 (UL44-45) and HV054 (UL46) necessary for recombination flanking two expression cassettes with the AAvV-1 HN gene under the EF-1a promotor and the AAvV-1 F gene under the ECMV promotor. Expression cassettes were inserted in intergenic non-coding sequence between HV053 (UL45) and HV054 (UL46) (Figure S1 in the Supplementary). The backbone recombinant vector pHV052-54 with homologous HVT sequences HV052-HV053 and HV054 containing required restriction sites was ordered from GeneCust. A double expression cassette was obtained by multistep cloning and was inserted into recombination plasmid pHV052-54 between HVT arms. The expression plasmids were sequenced and expression of F and HN genes was verified after transfection of CEF cells by in situ immunodetection (IPMA) using chicken anti-NDV serum (Figure S2 in the Supplementary). Cells transfected with plasmid carrying double expression cassetes coexpressing F and HN form multinucleated syncytia showing that F protein fusiogenic properties are activated by HN and are similar to highly virulent AAvV-1 strains.

### 3.2. Generation of Recombinant HVT-NDV-HN-F Virus

For generation of the recombinant HVT-NDV-HN-F virus, CEF cells infected with wt HVT were transfected with recombination transfer vectors containing double expression cassettes pHV-052-NDV-HN-F and also in parallel with pHV-052-F-EGFP. The recombination process was monitored by using recombination EGFP expressing plasmid pHV-052-F-EGFP (expressing also F protein of NDV LaSota) and IPMA using chicken anti-AAvV-1 sera (Figure S3 in the Supplementary). Recombinant HVT-NDV-F-HN infected cells were partly purified and concentrated by FACS and after few rounds of plaque purification (virus was purified by dilution and cocultivation of infected cells with fresh not infected cells in 96-well plates) (Figure S3). Virus was by propagated from a single plaque in quantities sufficient for animal experiments. Recombinant virus DNA was purified and verified by NGS sequencing (in house) to confirm presence of both F and HN expression cassettes.

### 3.3. Expression of HN and F Proteins in HVT-NDV-F-HN Infected Cells

Expression of both HN and F genes was confirmed by Western blotting using our in house prepared rabbit sera specific for F and HN proteins (Figure S4 in the Supplementary). On the same gel wt HVT lysate as a negative control was loaded. Bands for the F protein were visualized around 52 kDa corresponding to F1, a faint band around 66 kDa corresponding to F0 and a band around 110 kDa corresponding to the F1 dimer. For the HN protein a band around 75 kDa was detected.

### 3.4. Genetic Stability of Recombinant HVT-NDV-HN-F Virus

The recombinant virus titer remained unchanged at the level of 10^6.5^ TCID_50_ after each of tenth passages on CEF cells. Moreover, as shown in Figure S5 in the Supplementary, the presence of inserted HN and F genes as well as HVT genome fragments were basically on the same level.

### 3.5. Serological Response to Vaccination

The details about the dynamics of immune response measured by HI and ELISA are presented in Table 2. Briefly, at the time of challenge, the presence of antibodies was detected in 19/20 birds (HI & ELISA, Trial I), 5/20 (HI) to 7/20 (ELISA) birds (Trial II), and in 12/18 (HI) to 16/18 (ELISA) birds (Trial III).

### 3.6. Protective Efficacy of HVT-NDV-F-HN in SPF Chickens after Challenge with AAvV-1 Genotype IV: Trial I

The first detectable anti- AAvV-1 antibodies were found at 2 weeks of age by both HI test and ELISA test and then a sharp increase in antibody response was observed in the following weeks. At the time of challenge (6 wpv), 19/20 birds were positive by HI and ELISA tests (Table 2). A complete protection was observed following challenge with the velogenic strain of AAvV- administered 6 weeks p.v. since none of the SPF birds showed any clinical signs throughout the observation period (2 weeks) while all PBS-inoculated control birds died within 5 days post infection (Figure S6 in the Supplementary). Shedding of the challenge virus was detected only at day 3 p.c. (<10^3^eqEID50) in oropharyngeal swabs (15/20 birds) and cloacal swabs (1/20 birds). The difference in the amount of the excreted virus between experimental and control birds was statistically significant (*p* < 0.001).

### 3.7. Protective Efficacy of HVT-NDV-F-HN in Broiler Chickens after Challenge with AAvV-1 Genotype IV: Trial II

**Figure 1:**
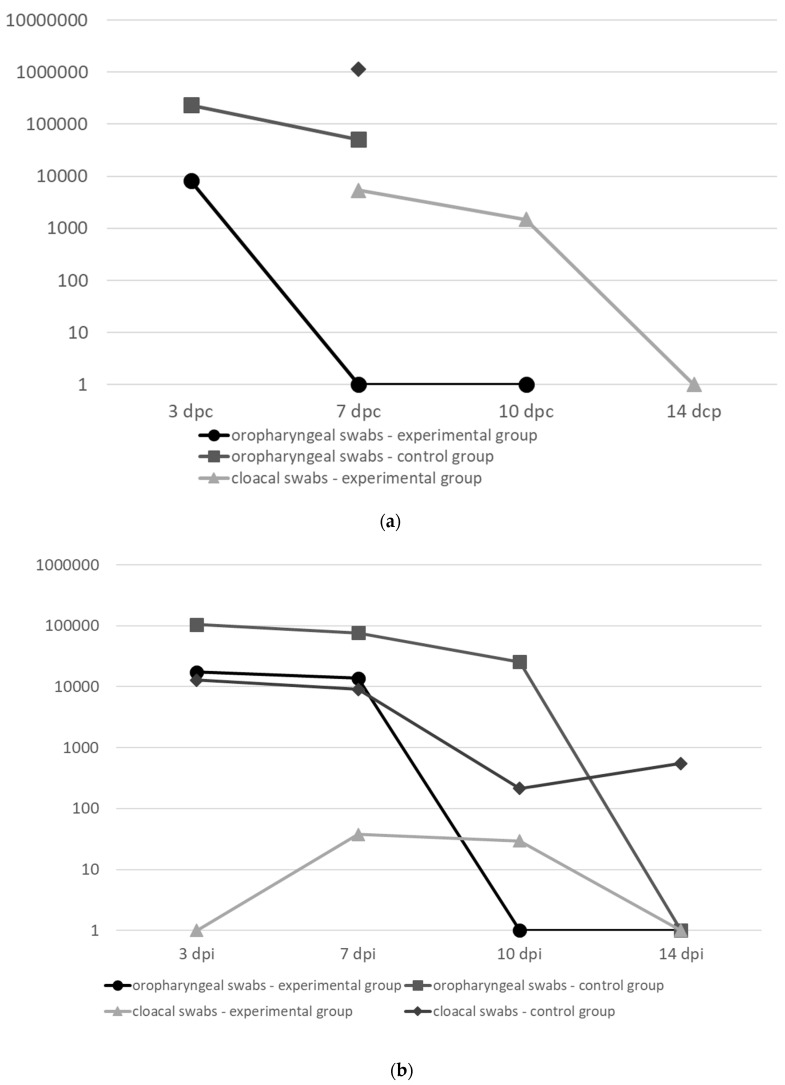
Median values of shedding of the challenge AAvV-1 virus of genotype IV (**a**) and genotype VII (**b**) in oropharyngeal and cloacal swabs of broiler chickens immunized with the HVT-NDV-F-HN construct at 1 day of age and challenged 6 weeks later expressed as eqEID_50_.

After challenge, no clinical signs were demonstrated in vaccinated/infected chickens until 7 days pc. At 7 dpc, 2 birds showed neurological signs and diarrhea and 1 bird died two days later while the second bird recovered. The remaining 18 chickens remained healthy until the end of experiment. All non-vaccinated and infected chickens became sick and died within 8 dpc (Figure S6). All surviving birds developed high levels of antibodies detected in the HI test (GMn = 8.3 log_2_) and ELISA (AMn = 16242). The presence of viral RNA was demonstrated at days 3, 7 and 10 pc in oropharyngeal swabs and at days 3, 7, 10 and 14 pc in cloacal swabs (Figure 1A). Because all control birds died by day 8 dpc, the statistical comparison of shedding between experimental and sham-inoculated groups was only performed for days 3 and 7 pc. All differences were statistically significant at *P* < 0.001 (oropharyngeal swabs at 3 dpc and cloacal swabs at 7 dpc) and *P* < 0.05 (oropharyngeal swabs 7 dpc).

### 3.8. Protective Efficacy of HVT-NDV-F-HN in Broiler Chickens after Challenge with AAvV-1 Genotype VII: Trial III

Two birds died during the first week of life and the cause of death was not established. After challenge, no clinical signs were demonstrated in vaccinated and infected chickens. Only at 6–7 dpc, some birds were less active but fed and drank normally. All non-vaccinated and infected chickens became sick and most of them (14/20) died within 9 dpc (Figure S6). All surviving birds developed high levels of antibodies detected in HI test (GMn = 7.2 log2) and ELISA (AMn = 24241). Viral RNA was detected at days 3 and 7 pc in oropharyngeal swabs and at days 7 and 10 pc in cloacal swabs (Figure 1B). Statistically significant differences were obtained between the experimental and control groups for cloacal samples at 3 dpc (*P* < 0.001), 7 dpc (*P* < 0.01), 10 and 14 dpc (*P* < 0.05) (Supplementary data).

## 4. Discussion

In this study we developed a recombinant vaccine using the HVT as a vector expressing the F and HN genes of AAvV-1 genotype VI and evaluated the humoral immune response following vaccination, the level of protection against clinical disease, mortality, as well as shedding of the challenge AAvV-1 strains representing genotypes IV and VII in SPF and commercial broiler chickens immunized subcutaneously at 1 day of age. The hypothesis behind the concept to produce a vector vaccine candidate expressing two immunogenic proteins was that the combination of both F and HN might induce a better protection than any of these glycoproteins alone. Kumar et al. showed that the F and HN proteins play a key role in the induction of protective immunity, although the contribution of F protein was shown to be greater than the HN protein [3]. However, there is little evidence on the protective value of an rHVT-F-HN against ND in chickens [22]. On the other hand, there is an abundance of scientific papers providing evidence for immunogenicity, safety and efficacy of a commercial HVT-based vector vaccine expressing solely the F gene and administered in ovo or at hatch in the presence of maternally derived antibodies. The commercialized vector vaccine, based on genotype I AAvV-1, proved to be successful against a variety of different genotypes including II, IV, V, VII and VIII [12,13,14,15].

In the present study, the HVT with an insert of two genes derived from the genotype VI AAvV-1 produced a stable recombinant construct. The genotype VI AAvV-1 is often referred to as “pigeon paramyxovirus type 1” (PPMV-1), because it is largely restricted in host range to birds of the family Columbidae [5]. However, infection of poultry with any virulent viruses of this genotype fall within the definition of Newcastle disease [23]. There is evidence that PPMV-1 was the source of ND outbreaks in poultry, including chickens and turkeys, in Great Britain, Germany, Sweden, Belgium or Estonia [23,24]. There is a paradigm that since all AAvV-1 strains belong to a single serotype, they should provide protection, at least against morbidity and mortality, caused by any AAvV-1 [14] and this was experimentally confirmed in a study where chickens immunized with AAvV-1 representing class I and class II viruses (genotypes I, II, V and VI) conferred >90% protection to clinical disease and mortality after challenge with virulent NDV [25]. Therefore, we decided to check the usefulness of the developed genotype VI-based construct HVT-NDV-F-HN as a potential vaccine candidate in chickens in a challenge study against a chicken-derived AAvV-1.

The first challenge AAvV-1 strain originated from our own repository. It belongs to genotype IV (“Herts-like”), that mostly comprise velogenic viruses responsible for the early epidemics of ND and were considered as predominant European NDV strains before 1970 [5,26]. The second AAvV-1 strain used for challenge was detected in late 1997 on a Swedish farm with broilers and layers and it was shown to belong to genotype VIIb [18]. Currently, genotype VII seems to be the most widespread in Asia, Africa, the Middle East and Europe although different sublineages are circulating in the field. The outbreaks of NDV in Europe since the early 1990s were typically caused by genotypes VIIa, VIIb, VIId and more recently VIIi genotypes [27]. The identification of almost unchanged viruses of VIIi genotype between 2011 and 2014 in such distant countries as Turkey, Bulgaria, Israel and Pakistan indicates how quickly the virus spreads causing health and economic problems in the poultry sector.

The developed HVT-NDV-F-HN construct was initially tested in SPF layer chickens (without maternal antibodies) immunized at 1 day of age via the subcutaneous route (trial I) and proved to be efficacious since neither clinical signs nor mortality were observed after challenge and virus shedding was significantly reduced.

A high level of clinical protection was achieved in broiler chickens despite a low humoral response rate observed, especially in the experimental trial II (Table 2). This phenomenon can be most likely explained by strong induction of the cell-mediated immunity caused by the fact that HVT replicates inside the lymphocytes and increases natural killer (NK) cell activity [28]. Moreover, a significant splenic cell-mediated immune response was shown in the study with HVT-ND vaccine expressing the F gene [29]. In a similar experiment with the rHVT-ND expressing the F gene in broilers, the ELISA titers were also below the threshold of positivity but the chickens remained protected against challenge with the virulent strain [13]. In another study performed in turkeys, the ELISA titers were positive at 6 weeks of age but the mean HI titers were below 2log_2_ [11]. Therefore, we conclude that the presence (and level) of antibodies has poor prognostic value for protection with the vector vaccine. Among the major advantages of the HVT-based vector vaccines is the ability to overcome maternally derived antibodies. However, the comparison of protection achieved in SPF chickens (MDA-negative birds) and broilers (MDA-positive birds) indicates that there is still limited interference of the vaccine with MDA.

In conclusion, our findings confirm that the HVT recombinant vaccine candidate expressing two genes (F and HN) derived from the genotype VI of AAvV-1 confers a broad clinical protection against heterologous challenge with velogenic chicken-derived AAvV-1 genotypes IV and VII and significantly reduces viral shedding in vaccinated birds. Among the major advantages associated with the application of the recombinant vaccine is the possibility of early vaccination of birds at the hatchery, limited interference of MDA, achievement of a uniform level of flock immunity, and the potential to detect infection in vaccinated flocks, e.g. by using an NP-based ELISA test. However, the exact mechanism of the observed protection is difficult to determine at the current stage. It is also too early to conclude unequivocally that the inclusion of an additional component (i.e. HN gene) to the construct HVT-ND-F is superior to the one-component construct (i.e. HVT-ND-F or HVT-ND-HN), although it is an attractive hypothesis. Undoubtedly, the additional experiments with the appropriate control groups have to be performed to obtain unequivocal confirmation of this hypothesis. Since the protection was not directly correlated with humoral response, detailed immunological studies on cell-mediated immunity should also be performed. Furthermore, additional investigations are also needed to optimize the protective dose, to assess the onset and duration of immunity as well as to evaluate protection following the in ovo route of administration.

## Figures and Tables

**Table 1:** Overview of the experimental design.

Week Post Vaccination		0	2	3	5	6	8
Trial N^o^	Group	Vaccination	Blood collection	Blood collection	Blood collection	Blood collection and challenge	Blood collection
Trial I^1^ (SPF chickens)	vaccinated	Yes	Yes	Yes	Yes	Yes	Yes
	mock	No	Yes	Yes	Yes	Yes	Yes
Trial II^1^ (broiler chickens)	vaccinated	Yes	No	Yes	Yes	Yes	Yes
	mock	No	No	Yes	Yes	Yes	Yes
Trial III^2^ (broiler chickens)	vaccinated	Yes	No	No	Yes	Yes	Yes
	mock	No	No	No	Yes	Yes	Yes

^1^ birds challenged with AAvV-1 genotype IV; ^2^ birds challenged with AAvV-1 genotype VII.

**Table 2:** The results of serological examination of sera collected from SPF and broiler chickens immunized with the HVT-NDV-F-HN vaccine candidate.

Week Post Vaccination	SPF Chickens (Trial I^1^)		Broilers (Trial II^1^)		Broilers (Trial III^2^)	
	HI (log_2_) Positive/Total (Geometric Mean Titer; Range)	ELISA Pos/Total (Mean Titer; Range)	HI (log_2_) Positive/Total (Geometric Mean Titer; Range)	ELISA Pos/Total (Mean Titer; Range)	HI (log_2_) Positive/Total (Geometric Mean Titer; Range)	ELISA Positive/Total (Mean Titer; Range)
0	nt	nt	20/20* (8.7; 7–10)	20/20* (17110; 14784–19182)	20/20* (7.9; 5–9)	20/20* (24240; 21556–26016)
2	3/20 (2.7; 2–5)	14/20 (2390; 3–6917)	nt**	nt	nt	nt
3	18/20 (3.9; 2–6)	19/20 (5082; 0–9847)	4/20 (3.0; 2–4)	18/20 (2297; 731–4036)	nt	nt
5	19/20 (5.5; 3–7)	19/20 (7517; 158–12556)	0/20 (2.1; 2–3)	2/20 (523; 135–1192)	1/18 (2.2; 2–4)	16/18 (3623; 632–10261)
6	19/20 (4.7; 3–7)	20/20 (9038; 890–14791)	5/20 (3.1; 2–6)	7/20 (1619; 36–9165)	12/18 (3.6; 3–5)	16/18 (9335; 125–15470)
8 (2 weeks after challenge)	20/20 (5.9; 4–10)	20/20 (14564; 10556–16917)	19/19 (8.3; 6–9)	19/19 (16242; 13321–17285)	18/18 (7.2; 6–9)	18/18 (24241; 19692–26824)

^1^ birds challenged with AAvV-1 genotype IV; ^2^ birds challenged with AAvV-1 genotype VII; * maternally derived antibodies; ** nt—not tested.

## Supplementary Materials

The following are available online at https://www.mdpi.com/1999-4915/11/9/784/s1: Figure S1. Schematic presentation of the construction of the recombinant HVT-NDV-HN-F. Recombination cassette contain HVT sequences (HV052-53 on the left hand side of expression cassettes and HV054 on the right hand side) for homologous recombination and two expression cassettes with AAvV-1/pigeon/332/05 F and HN genes: 1st containing HN gene (inserted under EF-1 promoter with BGH polyA) and 2nd containing F gene (inserted under ECMV promoter with SV40 polyA). Recombination was performed by transfection of HVT-infected CEF with recombination cassette DNA.; Figure S2: Control expression of NDV HN and F genes in chicken fibroblasts after transfection with expression plasmids used for obtaining recombination transfer vector pHV052-54., Figure S3: Detection of recombinant HVT plaques after transfection on HVT-wild-type (wt) infected CEF cells., Figure S4: Western blot analyses of coexpression of the F and HN proteins in CEF cells infected with HVT-NDV-HN-F., Figure S5: Effect of multiple passages on the presence of inserted genes fragments of AAvV-1 and HVT., Figure S6: Survival plots for protection of chickens vaccinated with HVT-NDV-F-HN against challenge with virulent AAvV-1 genotype IV (Trial I and II) and genotype VII (Trial III) and sham-inoculated control chickens based on Kaplan-Meier analysis; Supplementary data 1: Raw data of real-time reverse transcription polymerase chain reaction (RT-PCR) in oropharyngeal and cloacal swabs of broilers challenged with AAvV-1 genotypes IV and VII.
