# Supplementary material for: A Recombinant Turkey Herpesvirus Expressing F and HN Genes of Avian Avulavirus-1 (AAvV-1) Genotype VI Confers Cross-Protection against Challenge with Virulent AAvV-1 Genotypes IV and VII in Chickens
Source: Viruses. 2019 Aug 25;11(9):784. doi: 10.3390/v11090784 (PMC6784189; doi:10.3390/v11090784)
Supplement: Supplementary file 1 [file viruses-11-00784-s001.zip › Supplementary.docx]

**Figure S1.** Schematic presentation of the construction of the recombinant HVT-NDV-HN-F. Recombination cassette contain HVT sequences (HV052-53 on the left hand side of expression cassettes and HV054 on the right hand side) for homologous recombination and two expression cassettes with AAvV-1/pigeon/332/05 F and HN genes: 1st containing HN gene (inserted under EF-1 promoter with BGH polyA) and 2nd containing F gene (inserted under ECMV promoter with SV40 polyA). Recombination was performed by transfection of HVT infected CEF with recombination cassette DNA.

| 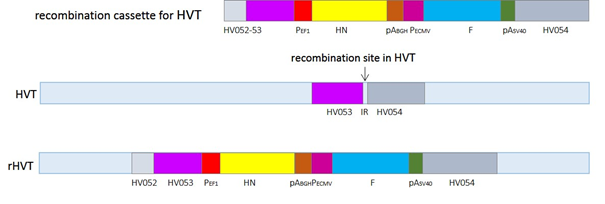 |  |
| --- | --- |

**Figure S2.** Control expression of NDV HN and F genes in chicken fibroblasts after transfection with expression plasmids used for obtaining recombination transfer vector pHV052-54. Immunodetection was performed by IPMA using mix of anti-NDV chicken sera. Observations were performed using confocal microscope.


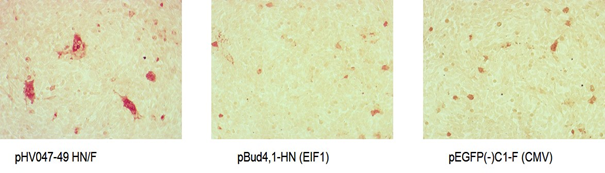


**Figure S3.** Detection of recombinant HVT plaques after transfection on HVT-wt infected CEF cells. Immunodetection was performed by IPMA with mix of chicken anti-NDV sera and confocal microscope observation in white light. GFP positive plaques were visualized also by UV light.


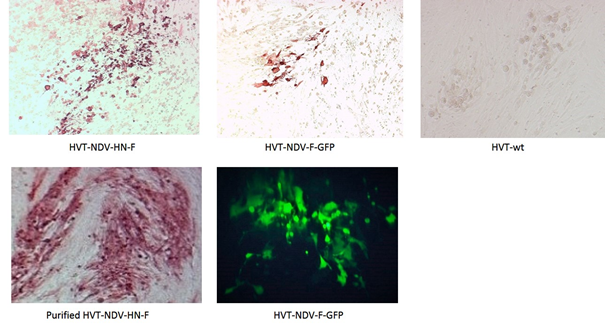


**Figure S4**. Western blot analyses of coxpression of the F and HN proteins in CEF cells infected with HVT-NDV-HN-F. CEF lysates in duplicate were subjected to 8% SDS-PAGE and electroblotted to PVDF membrane. Membrane was blocked and cut for separate detection of HN and F proteins with rabbit polyclonal sera specific for HN and F of NDV: AAvV-1 La Sota and AAvV-1/pigeon/332/05. Reaction was followed with incubation with alkaline phosphatase goat anti-rabbit IgG conjugate (Abcam). Protein bands were visualized by NBT/BCiP alkaline phosphatase substrate. The presence of both NDV proteins F and HN in samples is indicated (marked with arrows).


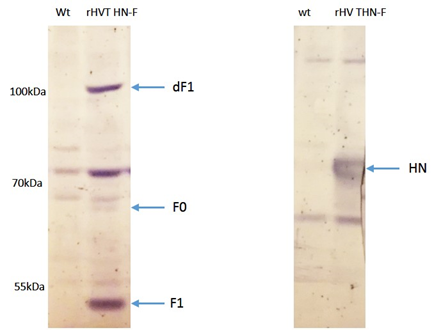


**Figure S5.** Effect of multiple passages on the presence of inserted genes fragments of AAvV-1 and HVT.


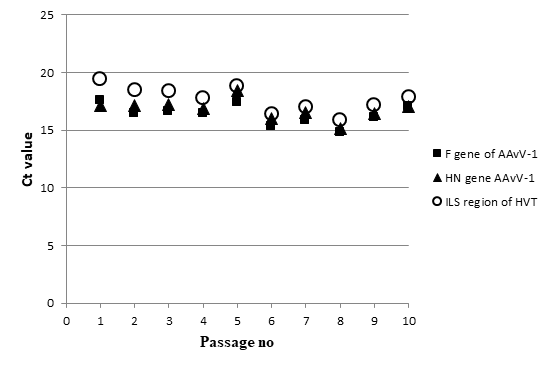


**Figure S6** Survival plots for protection of chickens vaccinated with HVT-NDV-F-HN against challenge with virulent AAvV-1 genotype IV (Trial I and II) and genotype VII (Trial III) and sham-inoculated control chickens based on Kaplan-Meier analysis. In each experiment the log-rank test showed significant difference in survival rate between vaccinated and control groups (p<0.0001)
