# Supplementary material for: A Recombinant Turkey Herpesvirus Expressing F and HN Genes of Avian Avulavirus-1 (AAvV-1) Genotype VI Confers Cross-Protection against Challenge with Virulent AAvV-1 Genotypes IV and VII in Chickens
Source: Viruses. 2019 Aug 25;11(9):784. doi: 10.3390/v11090784 (PMC6784189; doi:10.3390/v11090784)
Supplement: Supplementary file 1 [file viruses-11-00784-s001.zip › viruses-524030 final supplementary/Supplementary.pdf]

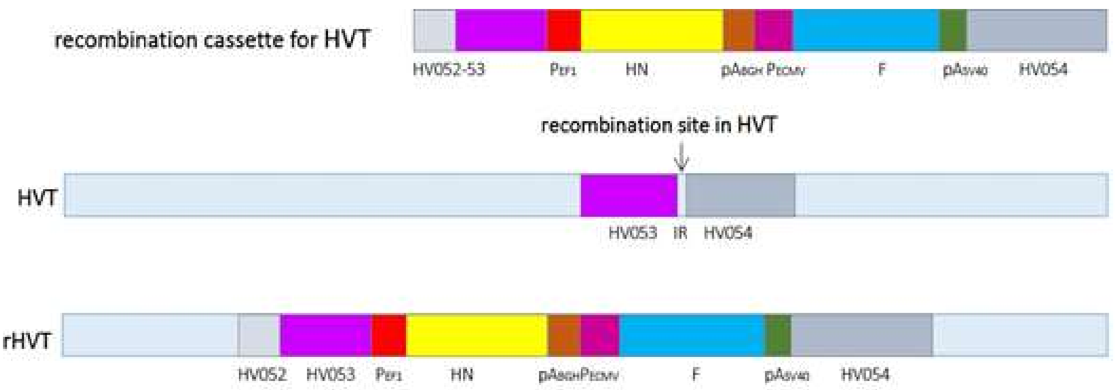

Figure S1. Schematic presentation of the construction of the recombinant HVT-NDV-HN-F. Recombination cassette contain HVT sequences (HV052-53 on the left hand side of expression cassettes and HV054 on the right hand side) for homologous recombination and two expression cassettes with AAvV-1/pigeon/332/05 F and HN genes: 1st containing HN gene (inserted under EF-1 promoter with BGH polyA) and 2nd containing F gene (inserted under ECMV promoter with SV40 polyA). Recombination was performed by transfection of HVT infected CEF with recombination cassette DNA.

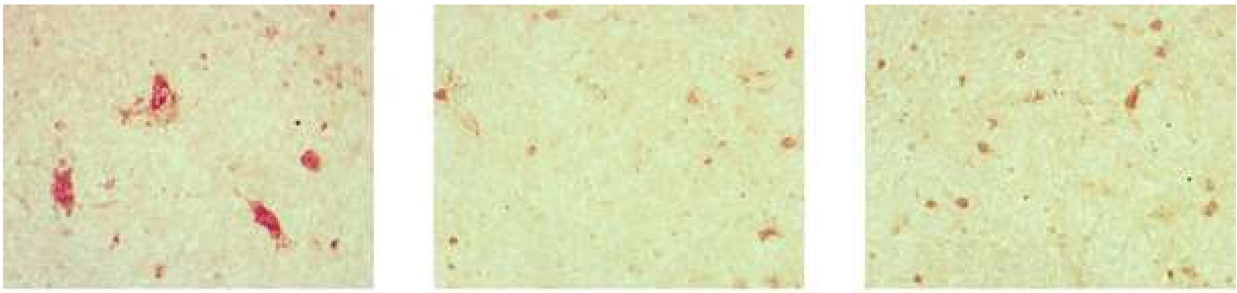

pHV047-49 HN/F | pBud4,1-HN (EIF1) | pEGFP(-)C1-F (CMV)

Figure S2. Control expression of NDV HN and F genes in chicken fibroblasts after transfection with expression plasmids used for obtaining recombination transfer vector pHV052-54. Immunodetection was performed by IPMA using mix of anti-NDV chicken sera. Observations were performed using confocal microscope.

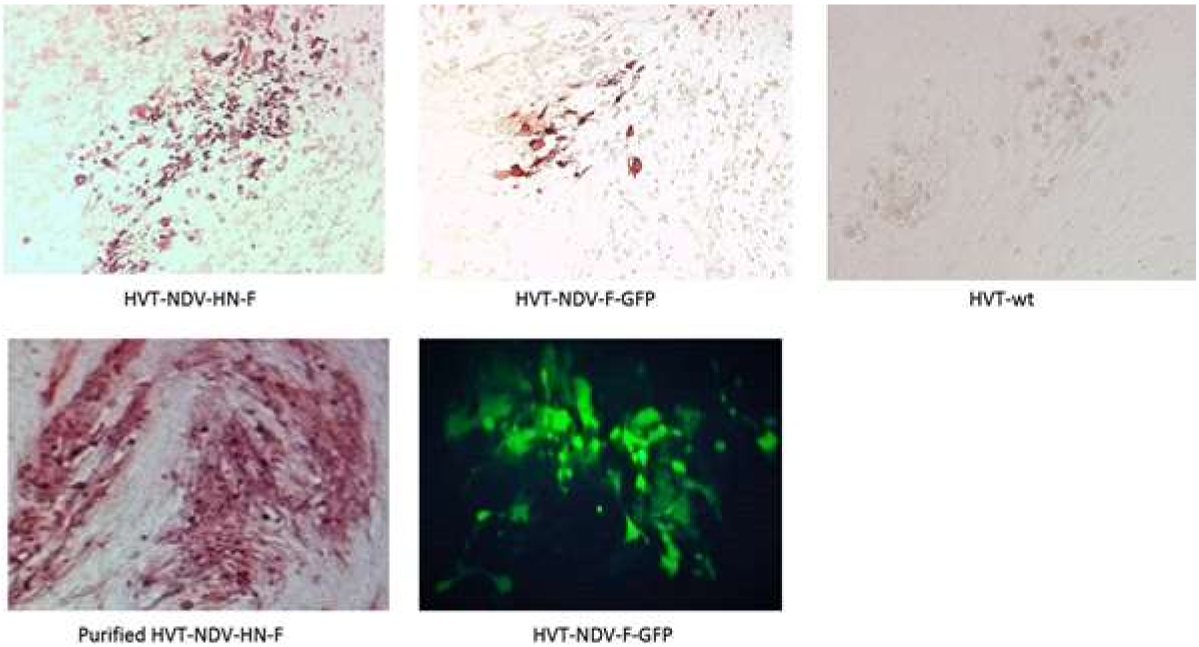

Figure S3. Detection of recombinant HVT plaques after transfection on HVT-wt infected CEF cells. Immunodetection was performed by IPMA with mix of chicken anti-NDV sera and confocal microscope observation in white light. GFP positive plaques were visualized also by UV light.

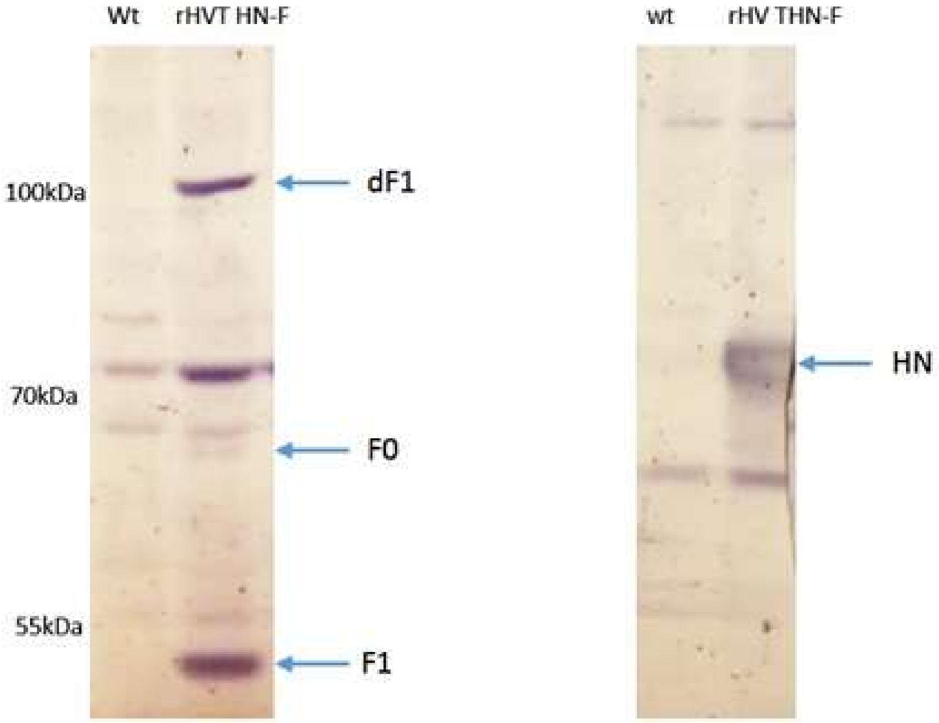

Figure S4. Western blot analyses of coxpression of the F and HN proteins in CEF cells infected with HVT-NDV-HN-F. CEF lysates in duplicate were subjected to 8% SDS-PAGE and electroblotted to PVDF membrane. Membrane was blocked and cut for separate detection of HN and F proteins with rabbit polyclonal sera specific for HN and F of NDV: AAvV-1 La Sota and AAvV-1/pigeon/332/05. Reaction was followed with incubation with alkaline phosphatase goat anti-rabbit IgG conjugate (Abcam). Protein bands were visualized by NBT/BCiP alkaline phosphatase substrate. The presence of both NDV proteins F and HN in samples is indicated (marked with arrows).

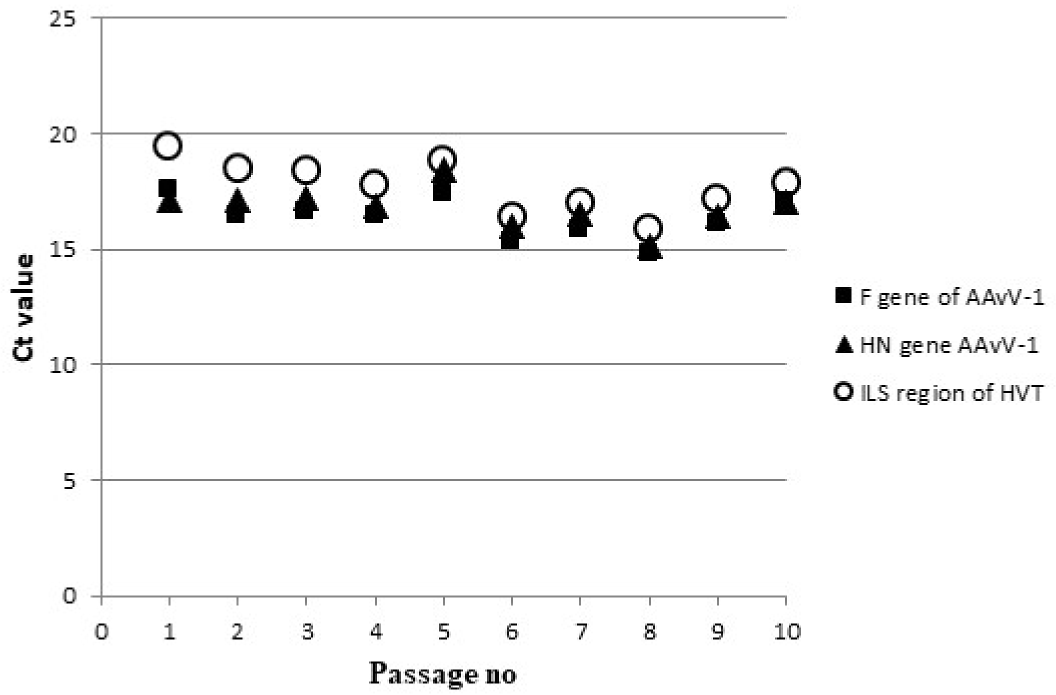

Figure S5. Effect of multiple passages on the presence of inserted genes fragments of AAvV-1 and HVT.

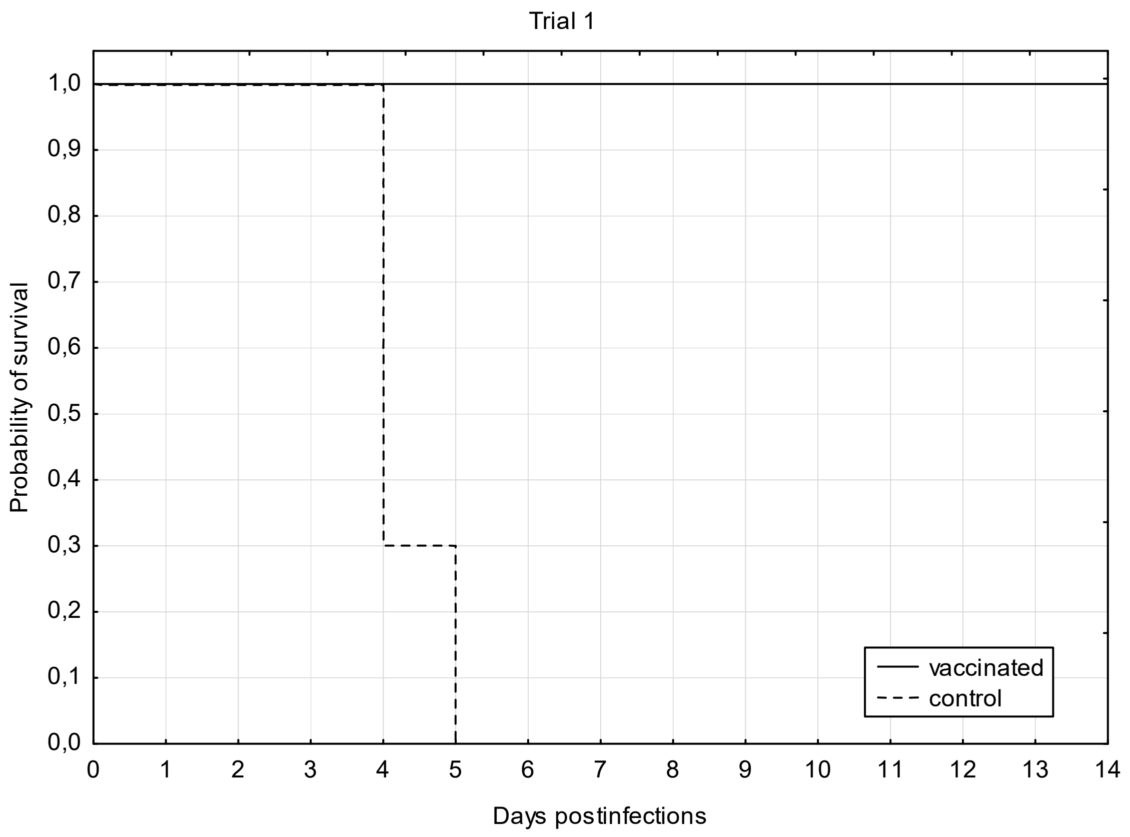
Trial 1
Probability of survival
Days postinfections
vaccinated
control
1,0
0,9
0,8
0,7
0,6
0,5
0,4
0,3
0,2
0,1
0,0
0 1 2 3 4 5 6 7 8 9 10 11 12 13 14

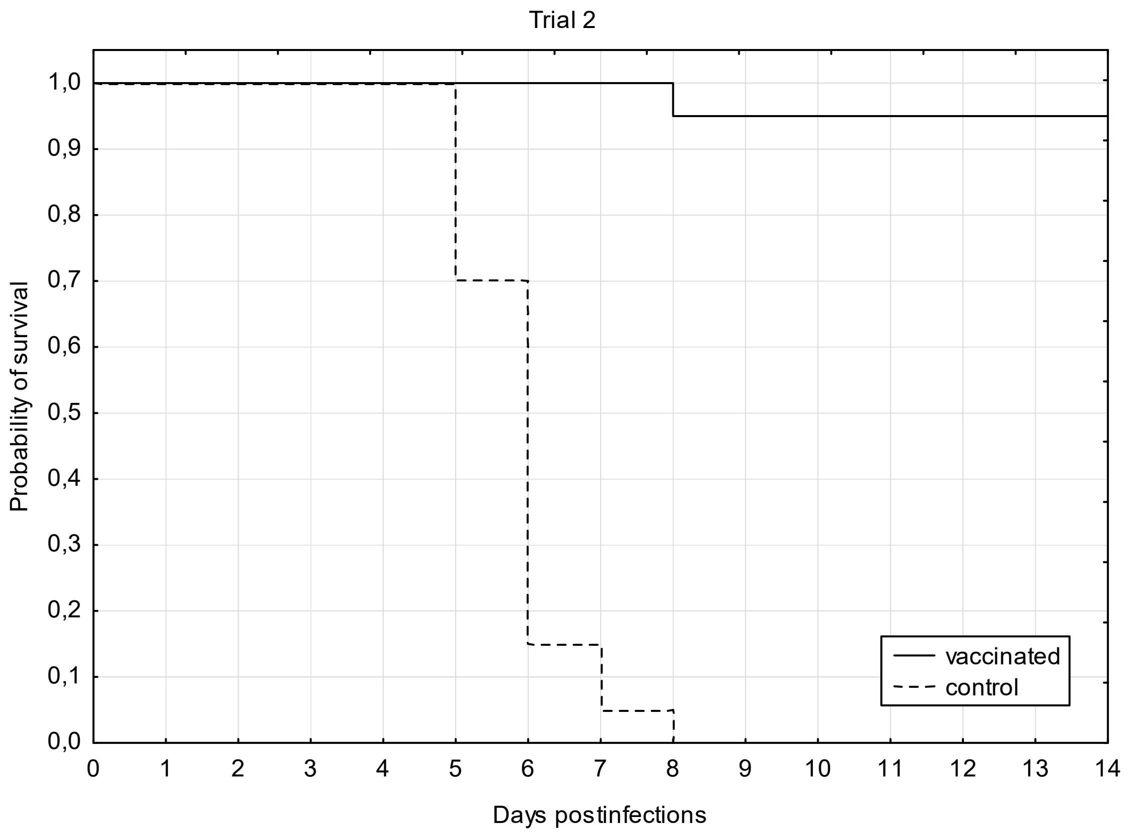
Trial 2
Probability of survival
Days postinfections
vaccinated
control
1,0
0,9
0,8
0,7
0,6
0,5
0,4
0,3
0,2
0,1
0,0
0 1 2 3 4 5 6 7 8 9 10 11 12 13 14

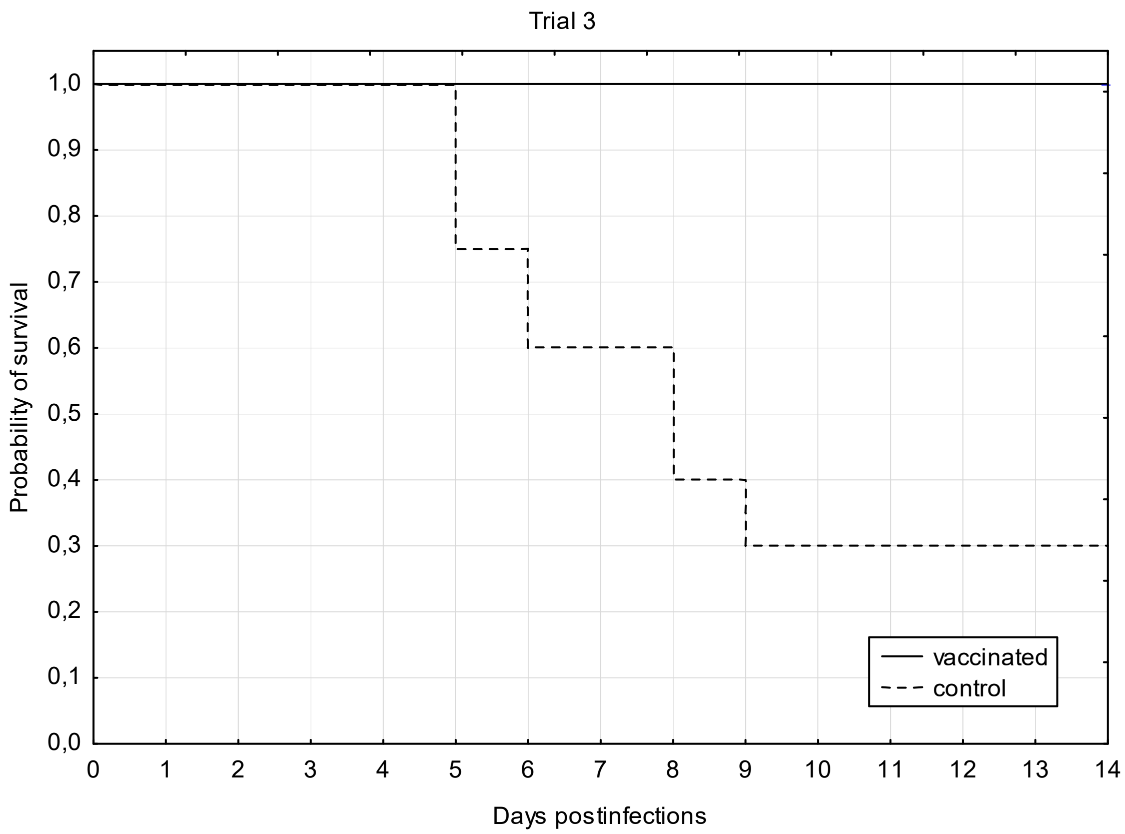

Figure S6 Survival plots for protection of chickens vaccinated with HVT-NDV-F-HN against challenge with virulent AAvV-1 genotype IV (Trial I and II) and genotype VII (Trial III) and sham-inoculated control chickens based on Kaplan-Meier analysis. In each experiment the log-rank test showed significant difference in survival rate between vaccinated and control groups ($p<0.0001$)
